# Potent Activities of Roemerine against *Candida albicans* and the Underlying Mechanisms

**DOI:** 10.3390/molecules201017913

**Published:** 2015-09-29

**Authors:** Chaoyu Ma, Faya Du, Lan Yan, Gonghao He, Jianchang He, Chengying Wang, Gaoxiong Rao, Yuanying Jiang, Guili Xu

**Affiliations:** 1 Department of Pharmacy, Kunming General Hospital of Chengdu Military Region, 212 Da-Guan Road, Kunming 650032, China; E-Mails: 15198808817@163.com (C.M.); faya_du@126.com (F.D.); hegonghao@163.com (G.H.); hejianchang123@sina.com (J.H.); 2 School of Pharmacy, Kunming Medical University, 1168 Yu-Hua Road, Kunming 650500, China; 3 New Drug Research and Development Center, School of Pharmacy, Second Military Medical University, Shanghai 200433, China; E-Mail: yl08101963@163.com; 4 School of Pharmacy, Yunnan College of Traditional Chinese Medicine, 1076 Yu-Hua Road, Kunming 650500, China; E-Mails: wcy19901111@126.com (C.W.); raogaoxiong@yeah.net (G.R.)

**Keywords:** antifungal activity, roemerine, *Candida albicans*, biofilm

## Abstract

Roemerine (RM) is an aporphine alkaloid isolated from the fresh rattan stem of *Fibraurea recisa*, and it has been demonstrated to have certain antifungal activity. This study aimed to investigate the antifungal activity of RM and the underlying mechanisms in *Candida albicans* (*C. albicans*). The *in vitro* antifungal activity of RM was evaluated by a series of experiments, including the XTT reduction assay, confocal laser scanning microscopy assay, scanning electron microscope assay. Results showed that 1 μg/mL RM inhibited biofilm formation significantly (*p* < 0.01) both in Spider medium and Lee’s medium. In addition, RM could inhibit yeast-to-hyphae transition of *C. albicans* in a dose-dependent manner. The biofilm-specific and hypha-specific genes such as *YWP1*, *SAP5*, *SAP6*, *HWP1*, *ECE1* were up-regulated and *EFG1* was down-regulated after 8 μg/mL RM treatment. Furthermore, the toxicity of RM was investigated using *C. elegans* worms, three cancer cells and one normal cell. The date showed that RM had no significant toxicity. In conclusion, RM could inhibited the formation of *C. albicans* biofilm *in vitro*, but it had no fungicidal effect on planktonic *C. albicans* cells, and the anti-biofilm mechanism may be related to the cAMP pathway.

## 1. Introduction

*Candida albicans* (*C. albicans*) is the most common fungal pathogen, which has a high propensity to cause superficial or life-threatening invasive infections in immunocompromised patients [1]. Although the need for effective antifungal therapy is increasing, the number of available antifungal agents is still limited. In addition, with the increasing clinical use of these limited antifungal agents, drug-resistant isolates are emerging rapidly [2].

A striking feature of *C. albicans* is its ability to grow in three morphological forms: budding yeast, pseudohyphal and hyphal forms [3]. Besides, *C. albicans* easily develops into a biofilm on the surfaces of almost any medical device, resulting in biofilm-associated infections [4]. Biofilms are structured microbial communities that are attached to a surface and are embedded in a matrix of exopolymeric material [5]. Comparing with planktonic cells, *C. albicans* biofilms display more severe resistance to a wide variety of clinical antifungal agents [6]. Therefore, new antifungal agents and strategies are urgently needed to address these challenges.

**Figure 1:**
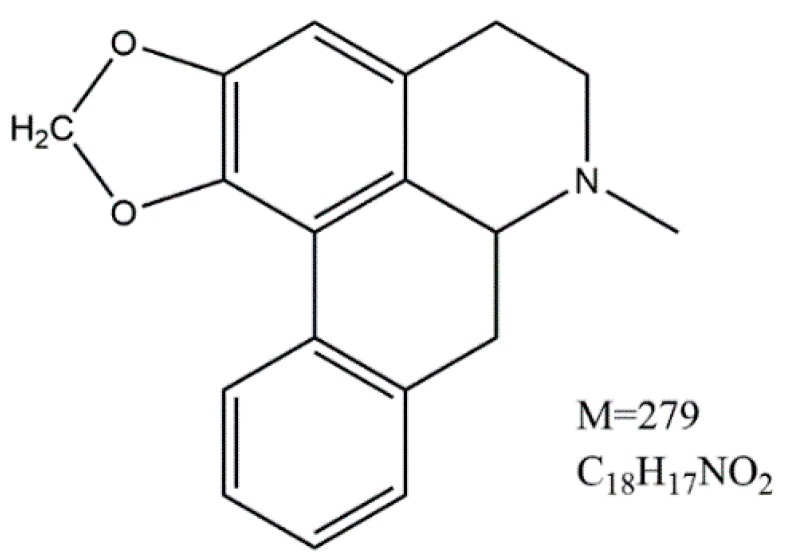
Chemical structure of RM, which is an aporphine alkaloid isolated from the fresh rattan stem of *Fibraurea recisa*.

Roemerine (RM) is an aporphine alkaloid (Figure 1). It is isolated from the fresh rattan stem of *Fibraurea recisa*. This bioactive alkaloid has a variety of pharmacological properties, such as vasodilator [7], anthelmintic [8] and antiplasmodial activities [9]. It also enhances the cytotoxicity of vinblastine against multidrug-resistant KB-V1 cells [10] and has high affinity for the 5-HT2A receptor [11]. In addition, it was reported that (+)-roemerine MeI, one of the derivatives of RM, had strong antibacterial activity against G+ bacteria, including *Bacillus cereus*, *Micrococcus* sp. and *Staphylococcus aureus* [12]. Besides, our previous studies showed that RM had a certain activity against fungal pathogens, such as *Candida albicans*, *Candida glabrata*, *Candida krusei*, *Candida parapsilosis*, and *Cryptococcus neoformans* [13]. Moreover, we also found that RM had great bioavailability (84%) in Sprague-Dawley (SD) rats, and it was suggested that RM could be taken orally instead of intravenously [14]. Based on this background, RM may be a promising drug against fungal pathogens. Development of RM as a new antifungal agent would be a significant advance in clinical treatment of fungus infection. Nevertheless, the effect of RM against *C. albicans* biofilms and the underlying mechanisms have not yet been investigated. 

In recent years, several signal transduction pathways like cyclic AMP (cAMP) pathway and mitogen-activated protein kinase (MAPK) pathway and some key transcription factors have been intensively investigated to uncover the mechanisms of *C. albicans* morphological transition. In these signal transduction pathways, some adhesion-, hypha-, and biofilm-specific genes, including *ECE1*, *RAS1*, *HWP1*, *YWP1*, *UME6*, *EAP1*, *ALS3*, *BCY1*, *EFG1*, *CSH1*, *CYR1*, *HGC1*, *CPH1* and *PDE2* play essential roles. Efg1 and Cph1 are transcription factors of cAMP and MAPK signaling pathways, respectively. However, Ras1 stimulates both the cAMP and MAPK pathways [15,16]. In this study, we evaluated the activities of RM against *C. albicans* biofilms *in vitro*, and exploring the underlying mechanisms, and we found that the antibiofilm mechanism is associated with the cAMP pathway.

## 2. Results

### 2.1. Effect of RM against C. albicans Yeast Cells

**Figure 2:**
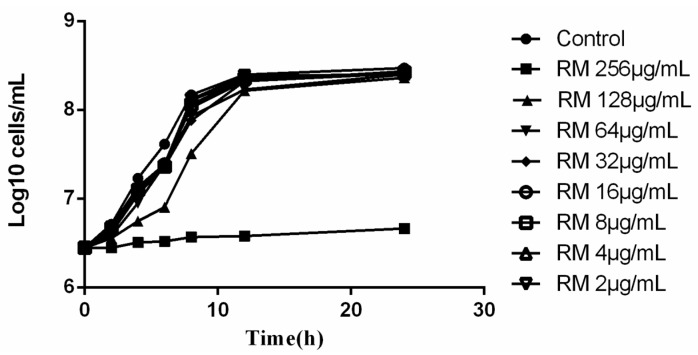
Effects of different concentrations of RM on fungal growth of *C. albican* SC5314. Exponentially growing *C. albicans* were suspended in YPD medium to the starting OD_600_ of 0.2 (about 2.5 × 10^6^ cells/mL). Then, the suspensions were added in different concentrations of RM and cultured at 30 °C, 200 rpm. *C. albicans* suspended in YPD medium with 1% DMSO was used as the control. Cells were counted at the predetermined time points (0, 2, 4, 6, 8, 12, 24 h).

The *in vitro* antimicrobial activity of RM was evaluated by broth microdilution assay, and the antimicrobial spectrum is demonstrated in Table 1. RM alone exhibited weak effect against *C. albicans* (MIC_80_: 128–256 mg/L), while it showed more potent activity against *Candida glabrata*, *Candida krusei*, *Candida tropicalis*, *Candida parapsilosis*, *Aspergillus fumigatus* and *Staphylococcus aureus* (Table 1). Meanwhile, the effect of different concentrations of RM on growth of *C. albicans* SC5314 was further established by time-growth curves (Figure 2). It showed that at concentrations ranging from 2 to 128 μg/mL, RM could not significantly inhibit the growth of *C. albicans*, the yeast cells growth was similar to that of the control group without RM treatment. By contrast, 256 μg/mL RM showed a significant effect on *C. albicans* growth (Figure 2).

**Table 1:** The antimicrobial spectrum of RM (MIC_50s_ and MIC_80s_ in mg/L).

Strains	RM (MIC_50s_, mg/L)	RM (MIC_80s_, mg/L)
*Candida albicans* SC5314	128	256
*Candida glabrata* 8535	32	64
*Candida krusei* 4996	16	32
*Candida tropicalis* 8915	32	64
*Candida parapsilosis* 90018	16	32
*Aspergillus fumigatus* 7544	16	32
* MRSA 135	32	64
MRSA 98	32	64
MRSA 310	16	32
MRSA 311	32	64
MRSA 111	32	64
MRSA 234	32	64
MRSA 92	32	64
MRSA 8	32	64
*Staphylococcus aureus* ATCC 25913	32	64

* Methicillin-resistant *Staphylococcus aureus*, MRSA.

### 2.2. RM Inhibits the Formation of C. albicans Biofilms 

**Figure 3:**
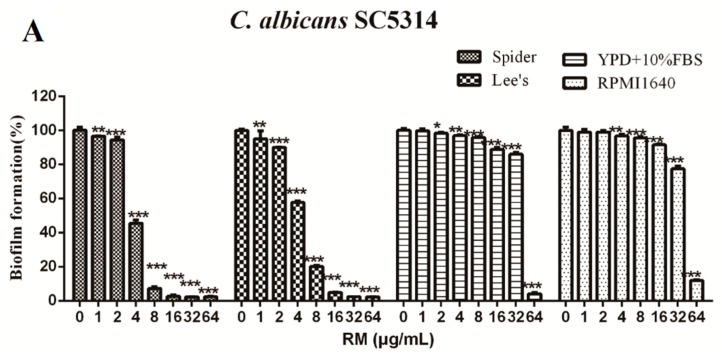

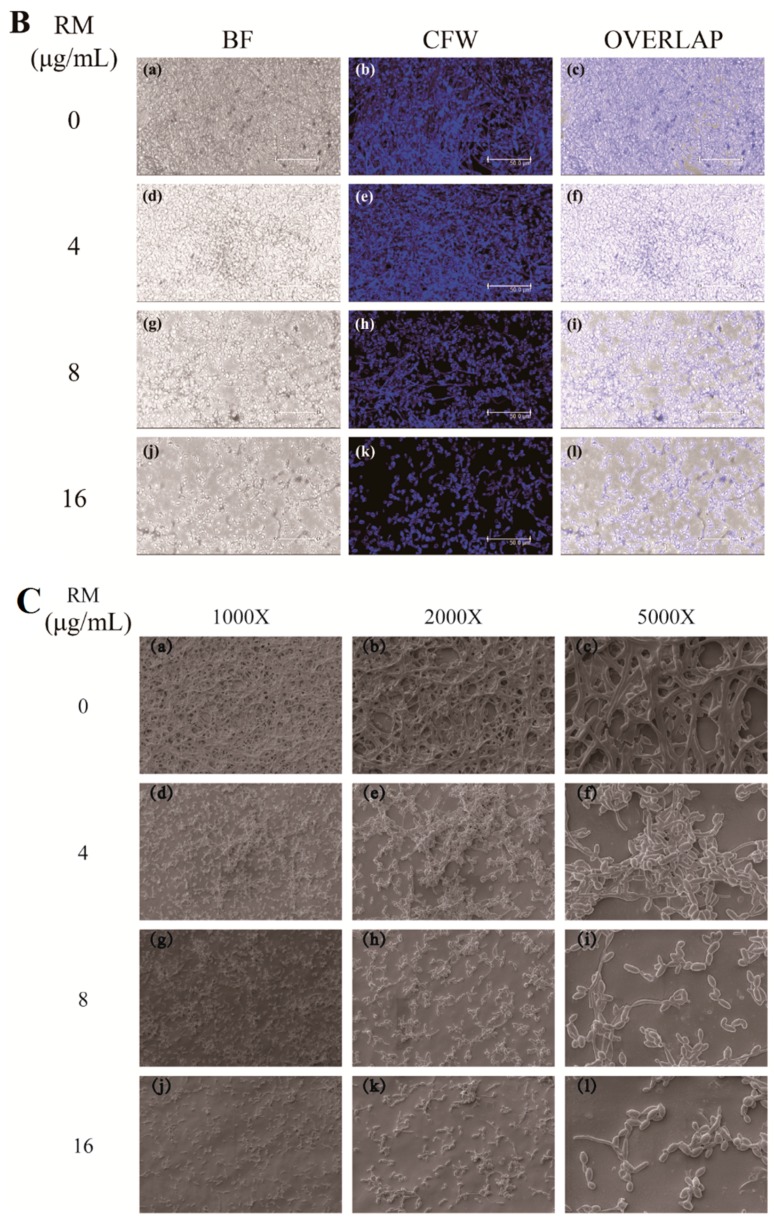
RM inhibits *C. albicans* SC5314 biofilm formation *in vitro*. (**A**) Inhibitory activity of different concentrations of RM on biofilm formation in four mediums (Spider, Lee’s, RPMI1640 and YPD + 10% FBS). * *p* < 0.05; ** *p* < 0.01; *** *p* < 0.001, compared to the control biofilms; (**B**) Effects of different concentrations of PTE on biofilm formation in Lee’s medium, shown in CLSM images. CFW, calcofluor white. Bar = 25 μm; (**C**) Effects of different concentrations of PTE on biofilm formation in Spider medium, shown in SEM images. The inset in the 1000×, 2000× and 5000× panels show the area that was magnificated. (**a**–**c**): normal biofilm; (**d**–**f**): cells were treated with 4 μg/mL RM; (**g**–**i**): 8 μg/mL RM; (**j**–**l**): 16 μg/mL RM.

To validate that RM has the anti-biofilm effect, we evaluated XTT reduction assay. In this study, we found that RM inhibited *C. albicans* biofilm formation in a dose-dependent manner (Figure 3A). In addition, one microgram per milliliter of RM inhibited biofilm formation significantly (*p* < 0.01) both in Spider medium and Lee’s medium. The anti-biofilm activity of 8 μg/mL RM in the Spider medium was the strongest and the *C. albicans* SC5314 biofilm formation was inhibited by 90% compared with the control. RM inhibited biofilm formation by 80% in the concentration in 8 μg/mL in Lee’s medium (Figure 3A). Furthermore, the anti-biofilm effect of RM was evaluated by CLSM (Figure 3B) and SEM (Figure 3C). *C. albicans* biofilm formation was disrupted by RM in a dose-dependent manner. In the drug free medium, *C. albicans* cells formed biofilm with a dense network of yeasts and true hyphae (Figure 3(Ba–c,Ca–c)). When cells exposed to 8 μg/mL RM, the biofilm formation was seriously disrupted (Figure 3(Bg–i,Cg–i)). Moreover, cell density was further reduced by 16 μg/mL RM (Figure 3(Bj–l,Cj–l)).

### 2.3. RM Inhibits the Yeast-to-Hypha Morphological Transition of C. albicans

**Figure 4:**
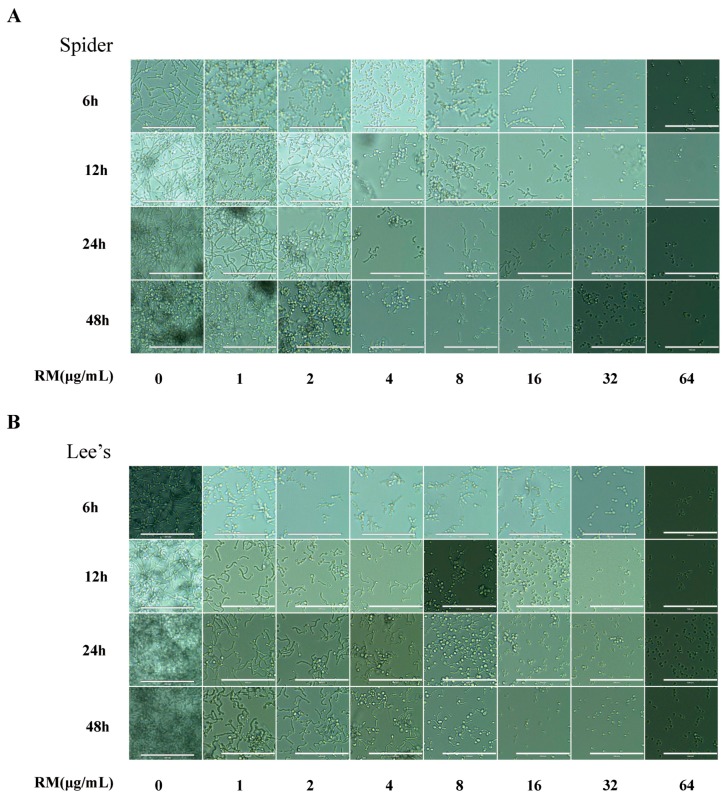

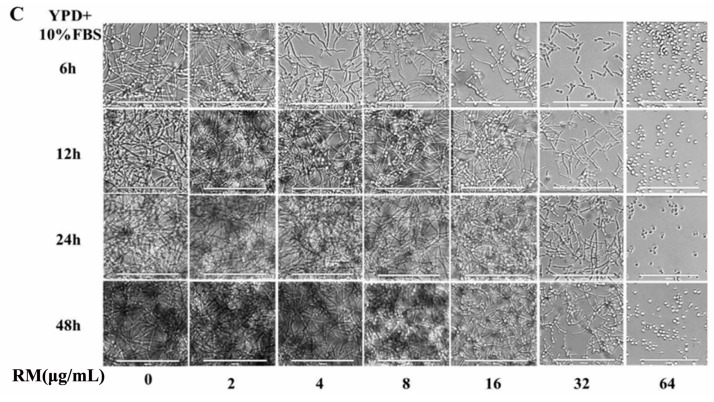
Effects of different concentrations of RM on hyphal formation. Exponentially growing *C. albicans* SC5314 cells were transferred to different hypha-inducing liquid media, (**A**) Spider medium; (**B**) Lee’s medium; (**C**) YPD + 10% FBS medium. The cellular morphology was photographed after incubation at 37 °C for 3.5 h. Bar = 100 μm.

To evaluate the effect of RM on the yeast-to-hypha morphological transition of *C. albicans*, three hypha-inducing mediums (liquid and solid media)—Spider, Lee’s, and YPD + 10% FBS—were used. RM showed strong hypha inhibition activity in different liquid medium (Figure 4A–C, Figure S1), the strong-to-weak sequence of the effect of RM against *C. albicans* biofilms was Spider medium, Lee’s medium and YPD + 10% FBS medium. In liquid Spider medium, one microgram per milliliter of RM could inhibit the growth of hypha, but the effect declined after 6 h, and this effect of RM on yeast-to-hypha transition enhanced with increasing RM concentrations. Four micrograms per milliliter of RM could inhibit the growth of hypha to 48 h, and 32 μg/mL RM could completely inhibit the hyphal growth (Figure 4A). In liquid Lee’s medium, one microgram per milliliter of RM could inhibit the growth of hypha, and the effect could continue after 48 h. Likewise, 32 μg/mL RM could completely inhibit the hyphal growth (Figure 4B). The *C. albicans* hyphal growth in liquid serum-containing medium was inhibited by 32 μg/mL RM (Figure 4C). Similarly, only smooth-edged colonies were observed on Spider (8 μg/mL) and Lee’s (2 μg/mL) solid medium (Figure 4D). When growing on serum-containing solid medium, colonies appeared smooth and round at 8 μg/mL, whereas, the colonies on the control plate were highly wrinkled and uneven (Figure S5). The results supported our hypothesis that RM could inhibit hyphal growth.

### 2.4. RM Decreases CSH of C. albicans Biofilm

**Figure 5:**
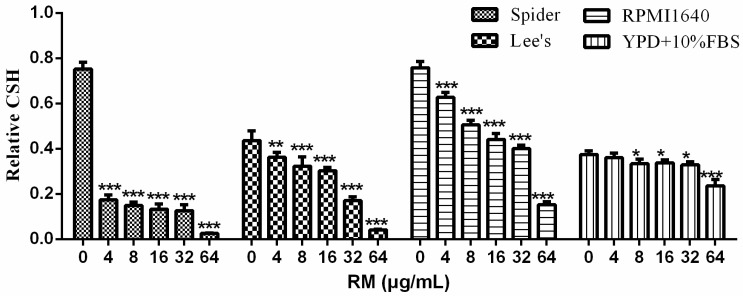
Effects of different concentrations of RM on CSH of *C. albicans* SC5314. The water-hydrocarbon two-phase assay was used to evaluate CSH. Standard deviations are based on three independent experiments. * *p* < 0.05; ** *p* < 0.01; *** *p* < 0.001.

In this study, the results showed that RM decreased CSH in a dose-dependent manner in four mediums (Spider, Lee’s, RMPI1640 and YPD + 10% FBS) (Figure 5). The effect in Spider medium was the strongest and 4 μg/mL RM significantly decreased CSH from 0.75–0.17 (*p* < 0.001).

### 2.5. Exposure to RM Alters C. albicans Gene Transcription

**Figure 6:**
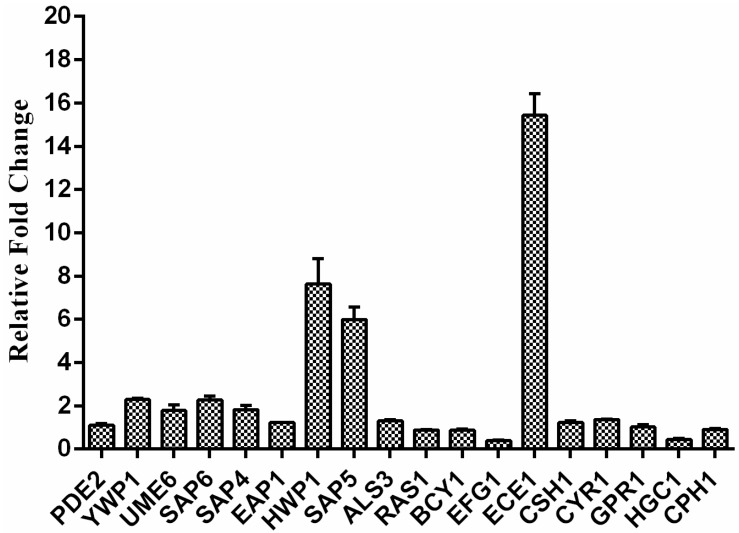
The expression changes of some important biofilm formation related genes after 8 μg/mL RM treatment. The *C. albicans* strain tested was SC5314. Gene expression was indicated as a fold change relative to that of the control group. 18S rRNA was used to normalize the expression data. Data are means ± standard deviations from three experiments.

In order to understand the anti-biofilm mechanism of RM, we further investigated the expression changes of genes after RM treatment using real-time RT-PCR. The result showed that the biofilm-specific and hypha-specific genes such as *YWP1*, *SAP5*, *SAP6*, *HWP1*, *ECE1* were up-regulated by 2.29, 5.99, 2.27, 7.63, 15.44-fold, respectively, and *EFG1* was down-regulated by 0.4013-fold after 8 μg/mL RM treatment (Figure 6). 

### 2.6. Exogenous cAMP Reverts the Morphogenesis Defect Caused by RM

**Figure 7:**
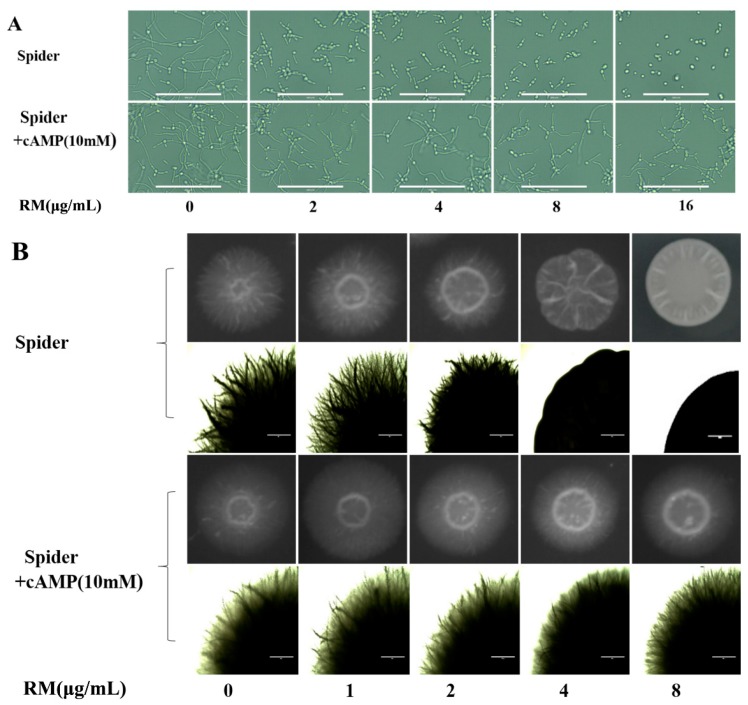
Addition of exogenous cAMP reverts the morphological transition defect of *C. albicans* SC5314 caused by RM. (**A**) Cells were incubated in liquid Spider medium supplemented with or without 10 mM cAMP. The cells were incubated at 37 °C for 4 h. Magnification 40×; (**B**) Hyphal formation on solid Spider medium plate with 10 mM cAMP and different concentrations of RM, the cells were incubated at 37 °C for 5 days.

Because some changed genes after RM treatment, such as *HWP1*, *ECE1* and *EFG1*, were regulated by Ras/cAMP pathway and cAMP/PKA pathway [17], we performed exogenous cAMP supplement assay to verify that the antibiofilm mechanism of RM was related to the cAMP pathway. The result showed that exogenous cAMP could revert the morphogenesis defect of *C. albicans* cells caused by RM (Figure 7A,B). When 10 mM cAMP were added in RM treated cultures, true hyphae were observed both in liquid and on solid Spider media (Figure 7A,B). This result indicates that RM may inhibit the biofilm formation by the cAMP pathway.

### 2.7. Cytotoxicity Studies 

In this study, the results showed that RM had good cytotoxic effect against cancer cell lines SGC-7901, HT-29 and MGC-803 with IC_50_ of 0.844 mg/L (3.02 µM), 1.279 mg/L (4.58 µM) and 0.631 mg/L (2.26 µM), respectively. Besides, RM had no significant cytotoxicity against human umbilical vein endothelial cell with the IC_50_ of 43.047 mg/L (154.11 µM). This experiment confirmed that RM had lower cytotoxic effect for non-cancer cell lines.

### 2.8. The Toxicity of RM on C. elegans Worms

**Figure 8:**
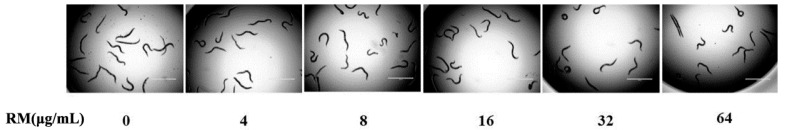
RM shows no significant toxicity on *C. elegans* glp-4; sek-1 nematodes. *C. elegans* glp-4; sek-1 nematodes were pipetted into 12-well plates that contained different concentrations of RM, incubated at 25 °C for 6 days, and observed daily.

We investigated the toxicity of RM using health adult *C. elegans* worms. The results showed that at a range of concentrations, from 4–32 μg/mL, RM did not display toxicity, and all the worms looked healthy (Figure 8). At 64 μg/mL RM, only two worms died, and so high concentration of RM exhibits low toxicity. Nevertheless, RM has certain toxicity for the worms, the LD_50_ of RM on *C. elegans* worms is 4096 μg/mL. One gram per milliliter of RM can make all the worms die.

## 3. Discussion

*C. albicans* is an opportunistic deep-infective fungi, still threatening our lives [18]. Most of established antifungal agents has been proved less than effective against *C. albicans* [19]. Therefore, the research and development of new antifungal agents is urgent. RM is a good therapeutic candidate that has been confirmed to have certain antifungal activity (MIC_80_: 160 mg/L) [13]. However, the effect of RM against *C. albicans* yeast cells and biofilm has not been elucidated. Consequently, in the current study, we focus on investigating the antifungal activity of RM against *C. albicans* biofilm and exploring the underlying mechanisms. 

In this study, we found that RM had strong antimicrobial activity against common pathogens, such as *A. fumigatus*, *S. aureus*, *C. glabratas*, *C. krusei*, *C. parapsilosis*, and *C. tropicalis*. Unfortunately, RM showed weak activity against *C. albicans* (MIC_80_: 128–256 μg/mL). *C. albicans* forms biofilms easily, which results in *C. albicans* resistance to traditional antifungal agents [20]. Biofilm formation includes three stages: adhesion to biomaterial surfaces, growth to form an anchoring layer, and morphological transition to form a complex three-dimensional structure. To validate that RM has an anti-biofilm effect, we evaluated XTT reduction assay. Of note, we found that RM had strong effect against *C. albicans* biofilm. RM could inhibit the growth of biofilms in different liquid mediums, the strong-to-weak sequence of the effect of RM against *C. albicans* biofilms was Spider medium, Lee’s medium and YPD + 10% FBS medium. Furthermore, CLSM assay and SEM assay were established to observe biofilm formation directly, and the results were highly consistent with that obtained by XTT reduction assay. In summary, RM has a strong antibiofilm effect against *C. albicans in vitro*.

It was demonstrated that biofilm formation and the yeast-to-hypha morphological transition contributed to the pathogenic potential of *C. albicans* [21]. Our data showed that RM significantly inhibited the yeast-to-hypha morphological transition in both liquid and solid medium. The results indicate that the anti-biofilm activity effect of RM may be involved the growth of *C. albicans* hypha rather than yeast cells. In this study, our results showed that 4 μg/mL RM decreased CSH significantly (*p* < 0.001). CSH is one of the major pathogenic attributes of *C. albicans* and showed a positive correlation with adhesion [22]. It is the indicator for adhesion ability. These results indicate that the anti-biofilm effect of RM seems attributable to its anti-adhesion and anti-morphological transition activities. 

In order to understand the anti-biofilm mechanism of RM, real-time RT-PCR assay was further performed. The data showed that the biofilm-specific and hypha-specific genes such as *YWP1*, *SAP5*, *SAP6*, *HWP1*, *ECE1* were up-regulated and *EFG1* was down-regulated after 8 μg/mL RM treatment. *ECE1* is a hypha-specific gene and it encodes a membrane protein dependent on the cAMP pathway [23]. *HWP1* is a specific hyphal gene encoding a cell wall mannose protein, which is essential for normal growth of the mycelium. It also plays an important role in the pathogenesis of Candida infections. Mutation of the *HWP1* gene results in incomplete biofilm formation [24]. *ECE1* and *HWP1* are regulated by Efg1. The protein is a transcription factor of the Ras/cAMP pathway and the cAMP/PKA pathway in hyphal development [17]. *SAP5* and *SAP6* encode secreted aspartic proteinase, which is expressed during hyphal growth. Expression of *SAP5* and *SAP6* also depends on Efg1 [25]. Based on this result, we speculated that the antibiofilm effect of RM might be related to the cAMP pathway. In this study, we fortunately found that exogenous cAMP could revert the hyphal morphogenesis defect of *C. albicans* cells caused by RM, the result further validated our speculation. However, the molecular mechanisms need to be further researched.

Fungal and mammalian cells belong to eukaryotic cells, thus, it is very difficult to research and develop a new antifungal drug without toxicity in mammals. In this study, we used healthy *C. elegans* worms to study the toxicity of RM, and the results indicated that at the final concentration as high as 64 μg/mL RM exhibited low toxicity. Cytotoxic activities of RM have been studied in lung adenocarcinoma cancer cells (A549), cervical adenocarcinoma cancer cells (HeLa), breast cancer cells (MCF-7), prostate cancer cells (LNCaP and PC3) and two human non-cancer cell lines: human embryonic kidney 293(HEK293) and neonatal foreskin fibroblast (NFF) cells [26]. Interestingly, RM had selective cytotoxicity against cancer cells, such as: A549 cells (IC_50_ of 3.4 µM) and HeLa cells (IC_50_ of 4.0 µM). However, RM had less cytotoxicity against non-cancer cell lines, HEK293 cells (IC_50_ of 15.9 µM) and NFF cells (IC_50_ of 15.2 µM), RM was selective against cancer cells because it had less inhibitory effect on the proliferation of non-cancer cells. It was noticable that the cells used in this study were not reported. The results revealed no significant cytotoxicity against human umbilical vein endothelial cell with the IC_50_ of 43.047 mg/L (154.11 µM). This experiment further confirmed that RM had lower cytotoxic effect for non-cancer cell lines although additional studies *in vivo* need to be carried out to determine the toxicity of RM in mammals.

## 4. Experimental Section 

### 4.1. Strains and Growth Condition 

In this study, the international general laboratory strain: *C. albicans* SC5314 [27], *Candida parapsilosis* 90018, *Staphylococcus aureus* ATCC25913 [28], three clinical *Candida* spp. isolates: *Candida glabrata* 8535, *Candida krusei* 4996, *Candida tropicalis* 8915, one clinical *Aspergillus fumigatus* isolates: *Aspergillus fumigatus* 7544, (Changhai Hospital, Shanghai, China) and eight clinical MRSA isolates (Kunming General Hospital of Chengdu Military Region, Kunming, China) were used. All strains were identified according to standard morphological criteria. Prior to test, strains grew on sabouraud dextrose agar (SDA) at 30 °C for 24 h and later subcultured in YPD (1% yeast extract, 2% peptone and 2% dextrose) liquid medium at 30 °C in a shaking incubator for 16 h. RM (purity ≥ 96.8%) was provided by Yunnan College of Traditional Chinese Medicine. It was dissolved in dimethyl sulfoxide (DMSO) for *in vitro* study.

### 4.2. Antifungal Susceptibility Testing

The minimal inhibitory concentration (MIC) was determined by the Clinical and Laboratory Standards Institute methods (M27-A3 and M27-S4) [29,30]. Briefly, the initial concentration in RPMI 1640 medium was 10^3^ CFU/mL for fungal suspension, and the final concentrations ranged from 4–256 μg/mL for RM. The 96-well microtiter plates were incubated at 35 °C for 24 h. The optical density at 630 nm (OD_630_) was measured to determine the growth inhibition. Each strain was tested in triplicate. The MIC_50_ was defined as the lowest concentration of the drugs that inhibited growth by 50% compared with the drug-free growth control.

### 4.3. Time-Growth Curves Assay

Exponentially growing *C. albicans* SC5314 were suspended in YPD medium to the starting OD_600_ of 0.2 (about 2.5 × 10^6^ cells/mL). Then the suspensions were added in different concentrations of RM and cultured at 30 °C, 200 rpm. Cells were counted at the predetermined time points (0, 2, 4, 6, 8, 12, 24 h). Three independent experiments were performed [31].

### 4.4. In Vitro Biofilm Formation Assay

The *in vitro* biofilm formation assay [32] was performed in a 96-well tissue culture plate by seeding 100 µL 1.0 × 10^6^ CFU/mL *C. albicans* cells suspension in Spider medium[33], Lee’s medium, YPD + 10% FBS (fetal bovine serum) [34] and RPMI 1640 medium and incubated at 37 °C. After 90-min adhesion, the medium was removed and the fresh medium with different concentrations of RM was added. And the plates were incubated statically at 37 °C for 24 h. Then the biofilms were washed with phosphate-buffered saline (PBS) and added 200 µL 2, 3-bis-(2-methoxy-4-nitro-5-sulfophenyl)-2H-tetrazolium-5-carboxanilide (XTT)/menadione. After incubating at 37 °C for 2 h in a dark room, the formed biofilms were determined using XTT reduction assay. 

### 4.5. Confocal Laser Scanning Microscopy (CLSM) Assay 

CLSM was performed to determine the inhibitory effect of RM on biofilm formation. The confocal laser scanning special culture dishes were inoculated with *C. albicans* 5314 at 37 °C for 90 min to allow adhesion. Then biofilms were stained with 100 μg/mL of calcofluor white (Fluka, 18909) in the dark. After that, samples were washed with 2 mL PBS and observed under a Leica TCS SP2 confocal laser scanning microscopy (excitation wavelength 355 nm; emission wavelength 433 nm) [35].

### 4.6. Scanning Electron Microscopy (SEM) Assay

For SEM assay, the biofilms were formed on silicone pads in Spider medium at 37 °C for 90 min [36]. After removing non-adherent cells, and the fresh medium with different concentrations of RM was added. Then, the samples were incubated at 37 °C for 24 h. Subsequently, biofilms were placed in fixed liquid specific for electron microscopy (G1020, Goodbio Technology CO., Ltd, Wuhan, China) for 2 h and dehydrated in an ascending ethanol series and dried naturally. The dry specimens were coated with gold and observed through an EVO MA1O SEM (Carl Zeiss GmbH, Eppelheim, Germany).

### 4.7. Yeast-to-Hyphae Transition Assays

Exponentially growing *C. albicans* SC5314 were incubated with different concentrations of RM in hypha-inducing liquid medium at 37 °C for 6, 12, 24, 48 h respectively and on solid medium(Spider, Lee’s, YPD + 10% FBS) at 37 °C for 5 days. The cells were observed and photographed by inverted phase contrast microscope (AMG^®^ EVOS xl, Life Technologies, Carlsbad, CA, USA).

### 4.8. Cellular Surface Hydrophobicity (CSH) Assay

CSH assay was based on water-hydrocarbon interface assay as described by Stephen A *et al.* [37]. The biofilms were harvested and suspended in YPD medium at OD_600_ of 1.0. A total of 1.2 mL of this suspension plus 0.3 mL octane was placed in a sterile glass tube and vortexed for 3 min. OD_600_ of the aqueous phase was recorded after settled for 10 min. OD_600_ of the suspension without the octane overlay was determined as control. Relative hydrophobicity (%) was obtained as [(OD_600_ of the control minus OD_600_ of the treatment)/OD_600_ of the control] × 100.

### 4.9. Real-Time RT-PCR

Real-time RT-PCR was performed according to the method described previously [38,39]. The biofilms of *C. albicans* 5314 were harvested and used for the total RNA extraction. Triplicate independent experiments were performed for each sample. The total RNA was extracted using Fungal RNAout kit (TIANDS, Beijing, China). 1st-strand cDNA was synthesized by reverse transcription reaction using a reverse transcription kit (TaKaRa, Biotechnology, Dalian, China). SYBR Green I (TaKaRa) and LightCycler Real-Time PCR system (Roche diagnostics GmbH, Mannheim, Germany) were used for Real-time PCR. Forward primer and reverse primer were listed in Supplementary Table S1. 18S rRNA played the role of internal control. The thermal cycling comprised four steps, an initial step at 95 °C for 2 min, 40 cycles at 95 °C for 10 s, 60 °C for 20 s, and 72 °C for 30 s. Finally, the gene expression level was calculated using the formula 2^−ΔΔCт^.

### 4.10. Exogenous Cyclic AMP (cAMP) Supplement Assay

Exponentially growing *C. albicans* SC5314 cells were resuspended in Spider liquid medium at 5.0 × 10^5^ CFU/mL. Then, different concentrations of RM with or without cAMP (10mM) were added. After that, the samples were incubated at 37 °C for 4 h. Simultaneously, 5.0 × 10^2^ CFU/mL *C. albicans* SC5314 cells were colonized on Spider solid medium at 37 °C for 5 days. The cells were observed and photographed by inverted phase contrast microscope (AMG^®^ EVOS xl). 

### 4.11. Cytotoxicity Studies

Three cancer cells (human gastric cancer cell line SGC-7901, human colon cancer cell line HT-29, human gastric cancer cell line MGC-803) and one normal cell (human umbilical vein endothelial cell) were employed in cytotoxicity tests. The cytotoxicity of RM was evaluated as the concentration of 50% cellular cytotoxicity (IC_50_) [40].

### 4.12. Toxicity Evaluation using C. elegans Worms

*C. elegans* glp-4; sek-1 adult nematodes were used to evaluate the toxicity of RM. Briefly, the nematodes were moved from *Escherichia coli* OP_50_ to pathogen-free liquid medium that contained different concentrations of RM or 1% DMSO. Then, the worms were incubated at 25 °C for 6 days and observed daily.

## 5. Conclusions 

In the present study, we found that RM obviously inhibits yeast-to-hypha transition and the *C. albicans* biofilm formation *in vitro*, but it has no fungicidal effect on planktonic *C. albicans* cells, and the anti-biofilm mechanism may be related to the cAMP pathway. In addition, RM is a safe alkaloid for human non-cancer cell lines. Whether the anti-biofilm effect of RM is applicable in clinical management requires further study, and *in vivo* activities of RM against *C. albicans* need to be investigated.

## Supplementary Materials

Supplementary materials can be accessed at: http://www.mdpi.com/1420-3049/20/10/17913/s1.
