# Supplementary material for: Potent Activities of Roemerine against Candida albicans and the Underlying Mechanisms
Source: Molecules. 2015 Sep 29;20(10):17913–28. doi: 10.3390/molecules201017913 (PMC6332056; doi:10.3390/molecules201017913)
Supplement: Supplementary file 1 [file molecules-20-17913-s001.pdf]

Supplementary Materials

Table S1. Primers used in the Real-Time RT-PCR assay.

Primer Name	Sequence
18S-F	TCTTTCTTGATTTTGTGGGTGG
18S-R	TCGATAGTCCCTCTAAGAAGTG
BCY1-F	TTGGAGGATTAGAAGCACTCT
BCY1-R	CACATCTGAATCACGACGAA
CPH1-F	TGCAGTTGCTACTACTGCTG
CPH1-R	CATGCTTTGATATCCCATGGC
CYR1-F	TGAGCCACCAATAGGAC
CYR1-R	AACGCATCACCTTCAGT
EAP1-F	TGTGATGGCGGTTCTTGTTC
EAP1-R	GGTAGTGACGGTGATGATAGTGACA
ECE1-F	GTATTCTTGGCAACATTCC
ECE1-R	ACGTCATCATTAGCTCCAT
EFG1-F	ACGTGGTAGAAGAGATGGGA
EFG1-R	TGCATTAGGAGTTACTCCGG
GPR1-F	AATGCTGCTGGTAATGG
GPR1-R	TGACTATGTCTCAGGGTA
HGC1-F	AACCACCACCACCAATGAA
HGC1-R	GAAACAGCACGAGAACCAG
HWP1-F	CAGAAGCTTCCATTCCACCT
HWP1-R	TTTGGAACAGCTGGAGAGGT
PDE2-F	ACCACCACCACTACTACTAC
PDE2-R	AAAATGAGTTGTTCCTGTCC
RAS1-F	GTTGTTGTTGGAGGTGGTGGTGTT
RAS1-R	GGCCAGATATTCTTCTTGTCCAGC
SAP4-F	CAATTTAACTGCAACAGGTCCTCTT
SAP4-R	AGATATTGAGCCCACAGAAATTCC
SAP5-F	CATTGTGCAAAGTAACTGCAACAG
SAP5-R	CAGAATTTCCCGTCGATGAGA
SAP6-F	CCTTTATGAGCACTAGTAGACCAAACG
SAP6-R	TTACGCAAAAGGTAACTTGTATCAAGA
YWP1-F	CTGATATTCGTAATGCTGGTAAAGTG
YWP1-R	GGAGTTTCACCCATTAATCTTCTTC
CSH1-F	CTGTCGGTACTATGAGATTG
CSH1-R	GATGAATAAACCCAACAACT
UME6-F	CCCATCATCAATCTTACCT
UME6-R	CACCACCAATAGAATCAAA

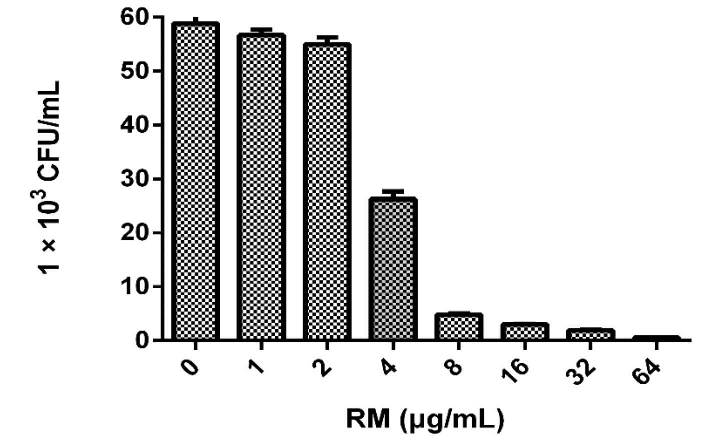

Figure S1. Effect of different concentrations of RM on biofilm formation of *Candida albicans (C. albicans)*. Exponentially growing *C. albicans* SC5314 were suspended in Spider medium at 1.0 × 10^6 CFU/mL, then 100 μL *C. albicans* cells were added in each well of a 96-well tissue culture plate. The samples were incubated at 37 °C. After 90 min, the medium was removed and the fresh medium with different concentrations of RM was added. The plates were incubated statically at 37 °C for 24 h. Then the biofilms were washed with phosphate-buffered saline (PBS) and suspended in Spider medium. Serial dilutions were plated on Spider agar to determine the CFU/mL of the live cells.

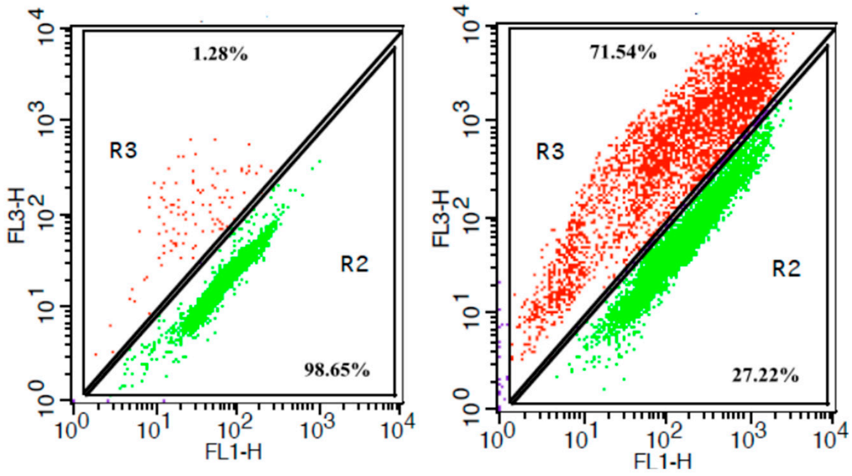

Figure S2. The experiment of live-dead staining of Candida cells treated with RM. The biofilms of *C. albicans* 5314 were formed in a 12-well tissue culture plate at 37 °C for 90 min. Then the medium was removed and the fresh medium with 8 μg/mL RM was added. Then the plates were incubated statically at 37 °C. After 24 h, the biofilms were harvested and washed thrice with PBS (0.01 M) and then resuspended in PBS buffer at 5 × 10^6 CFU/mL. Then, 498 μL of each sample were incubated with 1 μL SYTO-9 (3.34 mM) and 1 μL propidium iodide (PI, 20 mM) at room temperature for 30 min in the dark. Live cells were stained by the green-fluorescent stain SYTO-9 and dead cells were stained by the red-fluorescent PI stain. Then FACSCalibur flow cytometer (Becton Dickinson, San Jose, CA, USA) was used to distinguish live and dead *C. albicans* cells.

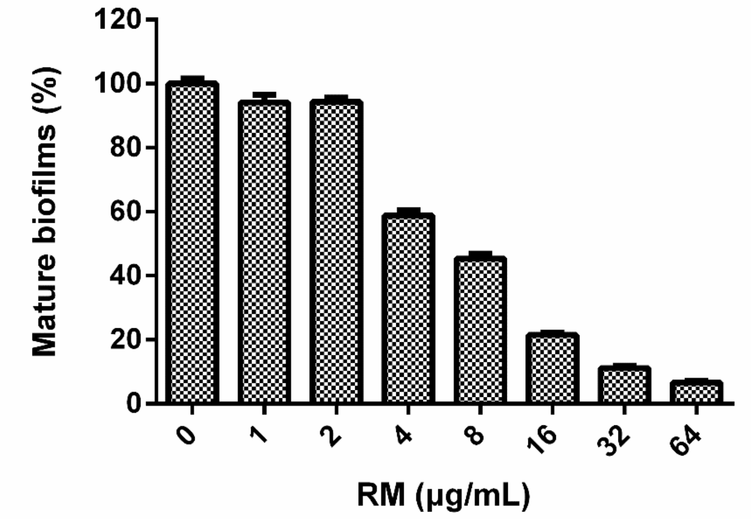

Figure S3. The effect of RM on *C. albicans* SC5314 mature biofilms. Exponentially growing *C. albicans* SC5314 were suspended in Spider medium at 1.0 × 10^6 CFU/mL, then 100 μL *C. albicans* cells were added in each well of a 96-well tissue culture plate. The samples were incubated at 37 °C. After 90 min, the medium was removed and the fresh medium was added. The plates were incubated statically at 37 °C for 24 h until formation of mature biofilms. Then the mature biofilms was determined using XTT reduction assay.

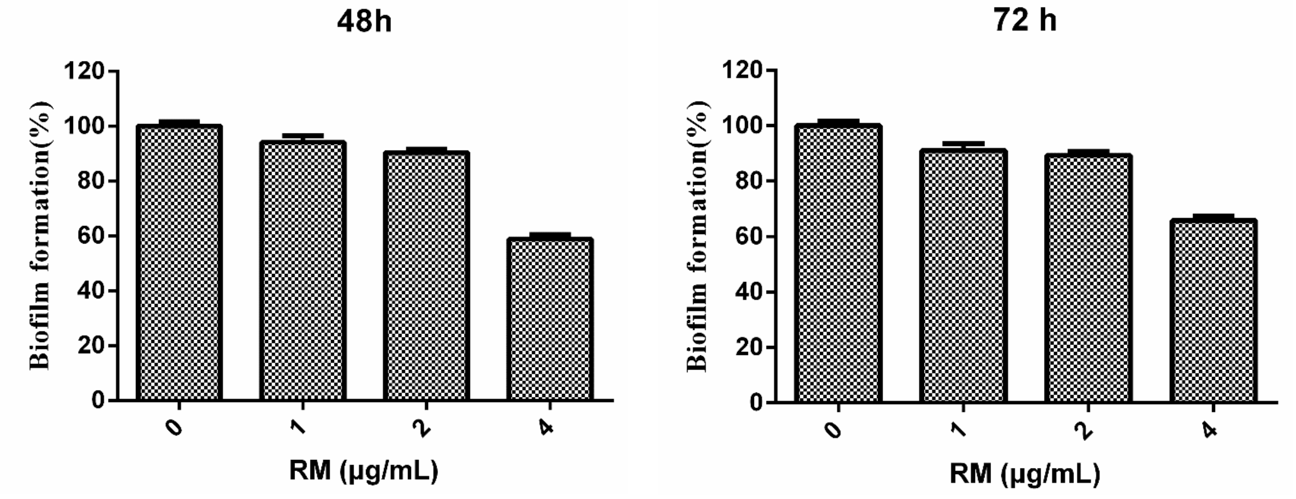

Figure S4. The effect of low RM concentrations on biofilms formation at 48 and 72 h.

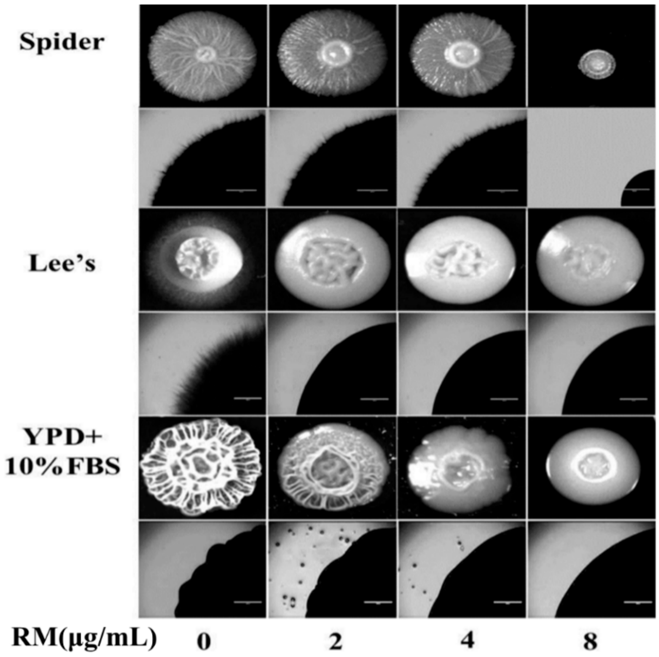

Figure S5. Effects of different concentrations of RM on hyphal formation. Exponentially growing *C. albicans* SC5314 cells were transferred to different hypha-inducing solid media, and incubated at 37 °C for 5 days. Colonies were photographed at ×4 magnification.

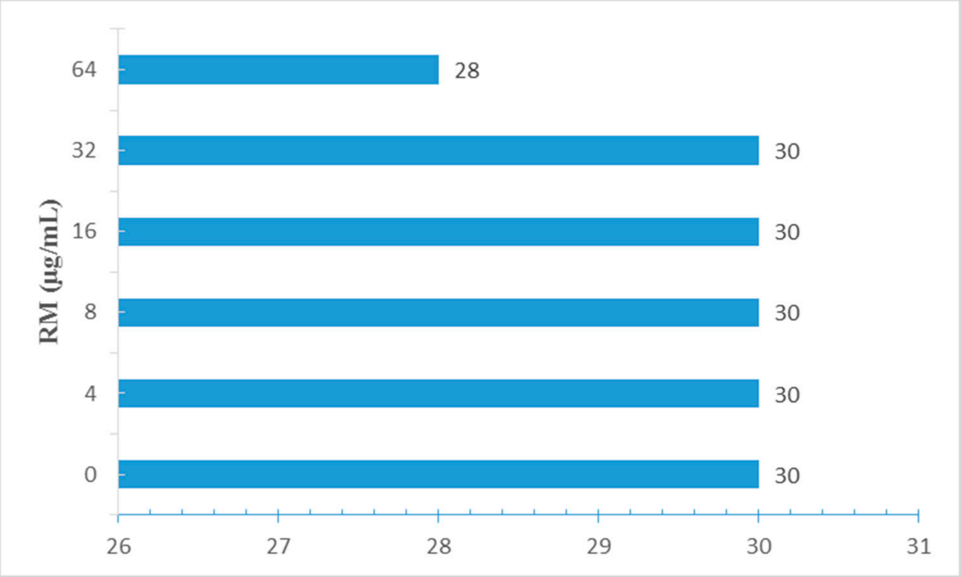

Figure S6. A bar graph of the toxicity of RM in worm model.
